# Residues of atrazine and diuron in rice straw, soils, and air post herbicide-contaminated straw biomass burning

**DOI:** 10.1038/s41598-024-64291-2

**Published:** 2024-06-10

**Authors:** Suteekan Lamnoi, Thirasant Boonupara, Sulak Sumitsawan, Patipat Vongruang, Tippawan Prapamontol, Patchimaporn Udomkun, Puangrat Kaewlom

**Affiliations:** 1 Department of Environmental Engineering, Faculty of Engineering, Chiang Mai University, Chiang Mai, 50200 Thailand; 2 School of Public Health, Environmental Health, University of Phayao, Phayao, 56000 Thailand; 3 Environmental and Health Research Group, Research Institute for Health Sciences, Chiang Mai University, Chiang Mai, 50200 Thailand; 4 Office of Research Administration, Chiang Mai University, Chiang Mai, 50200 Thailand

**Keywords:** Open burning, Soil pollution, Atmospheric pollutants, Biomass, Air pollution, PM_10_, Environmental sciences, Environmental impact

## Abstract

This study investigates the environmental impact of burning herbicide-contaminated biomass, focusing on atrazine (ATZ) and diuron (DIU) sprayed on rice straw prior to burning. Samples of soil, biomass residues, total suspended particulate (TSP), particulate matter with an aerodynamic diameter ≤ 10 µm (PM_10_), and aerosols were collected and analyzed. Soil analysis before and after burning contaminated biomass showed significant changes, with 2,4-dichlorophenoxyacetic acid (2,4-D) initially constituting 79.2% and decreasing by 3.3 times post-burning. Atrazine-desethyl, sebuthylazine, and terbuthylazine were detected post-burning. In raw rice straw biomass, terbuthylazine dominated at 80.0%, but burning ATZ-contaminated biomass led to the detection of atrazine-desethyl and notable increases in sebuthylazine and terbuthylazine. Conversely, burning DIU-contaminated biomass resulted in a shift to 2,4-D dominance. Analysis of atmospheric components showed changes in TSP, PM_10_, and aerosol samples. Linuron in ambient TSP decreased by 1.6 times after burning ATZ-contaminated biomass, while atrazine increased by 2.9 times. Carcinogenic polycyclic aromatic hydrocarbons (PAHs), including benzo[a]anthracene (BaA), benzo[a]pyrene (BaP), and benzo[b]fluoranthene (BbF), increased by approximately 9.9 to 13.9 times after burning ATZ-contaminated biomass. In PM_10_, BaA and BaP concentrations increased by approximately 11.4 and 19.0 times, respectively, after burning ATZ-contaminated biomass. This study sheds light on the environmental risks posed by burning herbicide-contaminated biomass, emphasizing the need for sustainable agricultural practices and effective waste management. The findings underscore the importance of regulatory measures to mitigate environmental contamination and protect human health.

## Introduction

The dry season in Southeast Asia, which spans from January to April, presents a significant environmental and health challenge due to air pollution^1^. The problem is exacerbated by widespread uncontrolled open burning of agricultural residues and an increase in forest fires, particularly in Chiang Mai Province and other Northern Thai regions^2,3^. Specifically, rice residue burning, a common practice among Thai farmers, is believed to benefit crop yields but has detrimental environmental consequences, notably in terms of air pollution and climate change^4^. Numerous studies consistently emphasize the crucial role of vegetative burning as a major contributor to overall particulate matter (PM) concentrations in ambient air^3,5–7^. Interestingly, when pesticides or harmful substances adsorb onto PM, they can be carried over extended distances by the wind, posing a heightened risk to distant populations through inhalation^8^. Reisen and Brown^9^ demonstrated that widespread burning of pesticide-treated biomass produces mixtures of toxic gases and smoke affecting populated areas. Air samples collected at forest fire sites indicated the release of pesticides and toxic combustion products, along with regional atmospheric transport of air pollutants resulting from biomass burning^10^.

The pollution issue in the region is exacerbated by prevalent agricultural, industrial, and anthropogenic activities, leading to a continual rise in micropollutant concentrations. Herbicides, notably atrazine (ATZ) and diuron (DIU), play a significant role in this environmental concern due to their extensive use in weed control in Thai agriculture^11^. ATZ and DIU disrupt both photosystem II and I in plants and photosynthetic microorganisms by inhibiting the chloroplast electron transport chain^12^. These herbicides exhibit high mobility within soil and aquatic environments due to their water-soluble characteristics and polar functional groups^13,14^. They are frequently detected in surface waters, underground reservoirs, and well water sources^15^.

Although there are currently no studies available on the distribution of ATZ and DIU in agricultural residues, their presence in the environment, including soils, water, and plants have been documented. The residual amounts of ATZ and DIU in soil can undergo sorption processes or migrate into water bodies directly through runoff or indirectly through leaching and erosion processes, particularly affecting agricultural regions^16^. This mobility has been demonstrated in studies such as Vonberg et al.^17^, which analyzed ATZ soil core residues from an agricultural field where ATZ had been applied before its ban in 1991, revealing an ATZ half-life value of about 2 years for the soil zone, significantly exceeding the highest ATZ half-lives found in the literature (433 days for subsurface soils). Similarly, Meng et al.^18^ observed the detection of ATZ in soil and surface water from 2014 to 2020, while hydroxyatrazine was found in soil without the selected analytes detected in groundwater. Additionally, ATZ bioaccumulation and biodegradation have been reported in some plants such as rice^19^ and cattail^20^. For DIU, Giacomazzi and Cochet^21^ indicated that DIU was more subject to leaching in deeper soil due to the small amount of organic matter, leading to easier pollution of water.

Furthermore, the presence of ATZ and DIU in agriculture poses significant risks to both human and environmental health. These herbicides can enter the body through inhalation and ingestion^22^, affecting various physiological systems^23–26^. ATZ disrupts the reproductive, excretory, and nervous systems, along with impacting plant photophosphorylation and oxidative stress markers^27–30^. While DIU leads to DNA fragmentation, urothelial cell proliferation, and urinary bladder issues^12,31,32^. It also has carcinogenic effects in rats and affects the reproductive systems of various organisms^33,34^. Interaction of DIU with other pesticides has also been shown to lead to varying toxic responses in invertebrates^35^.

Thailand's heavy reliance on herbicides and the widespread practice of biomass burning have raised significant concerns among researchers. This interconnected challenge demands immediate attention and a comprehensive understanding due to its potential impacts on the well-being of local populations and surrounding ecosystems. While many studies have investigated the presence of herbicides such as ATZ and DIU in soil and water, none have thoroughly explored their potential contributions to soil, ash, and atmospheric pollution during the open burning of herbicide-laden agricultural residues. Therefore, this study aims to fill this crucial knowledge gap by investigating the residues of ATZ and DIU post-biomass burning. The main objective is to evaluate the impact of burning herbicide-contaminated straw biomass on residue levels in rice straw, soils, and the surrounding air. Additionally, a comparative analysis has been conducted to assess the distinct impacts of ATZ and DIU. In Thailand, pesticides, especially herbicides, are often sprayed shortly before post-harvesting. Farmers may sometimes overlook withdrawal periods, leading to the detection of parent pesticides in their original form in significant quantities, as demonstrated in prior research. This motivated the design of the experiment, where the suggested application rate was applied to replicate a worst-case scenario involving residual biomass after harvesting.

## Materials and methods

### Raw material and chemicals

This study utilized rice straw sourced from a local farm area in Sanpatong district, Chiang Mai, Thailand, as the biomass material for open burn testing. ATZ and DIU herbicides, procured from local markets (ICP Ladda Co., Ltd., Bangkok, Thailand, and Khowtongseang Co., Ltd., Pichit, Thailand, respectively), were employed in the experiments. The chemical composition of herbicide solutions, with ATZ or DIU as the major constituents, is detailed in Table 1, and their molecular structures are provided in Table 2. Solvents from RCI Labscan (Bangkok, Thailand) were used for extraction and analysis. The Quick, Easy, Cheap, Effective, Rugged, and Safe (QuEChERS) Extraction Kit (containing magnesium sulfate, sodium chloride, sodium citrate, and disodium citrate sesquihydrate) and 2 mL of QuEChERS dispersive solid-phase extraction (SPE) with primary secondary amine (PSA), octadecysilane end-capped, and magnesium sulfate (Agilent, California, USA) were employed for purification.

**Table 1:** Composition of commercial herbicides atrazine and diuron analyzed using LC–MS/MS.

Chemical substance	Composition (%)	
	Atrazine	Diuron
Atrazine	93.41	0.09
Diuron	–	99.90
Propazine	3.12	–
Terbuthylazine	2.65	–
Simazine	0.76	–
Deisopropylatrazine	0.04	–
Atrazine-desethyl	0.01	–
2,4-D	0.01	0.01

**Table 2:** Experimental parameters for LC–MS/MS analysis of each herbicide.

Compound	Molecular formula	Chemical structure	Retention time (min)	Precursor ion (m/z)	Product ion (m/z)	Collision Energy (–)	Fragmentor (–)
2,4-D	C_8_H_6_Cl_2_O_3_		8.87	220.96	162.95	15	90
Atrazine	C_8_H_14_ClN_5_		9.31	216.1	62.1	56	125
			9.31	216.1	43.1	48	125
Atrazine-desethyl	C_6_H_10_ClN_5_		6.14	188.07	68	45	121
			6.14	188.07	62	45	121
Cyanazine	C_9_H_13_ClN_6_		7.31	241.1	104	44	120
			7.31	241.1	68	40	120
Deisopropylatrazine	C_5_H_8_ClN_5_		10.85	174.1	71.1	40	220
			10.85	174.1	62	28	220
Diuron	C_9_H_10_Cl_2_N_2_O		9.63	233.03	133	44	110
			9.63	233.03	46.1	15	110
Linuron	C_9_H_10_Cl_2_N_2_O_2_		10.52	249.02	133	36	100
			10.52	249.02	61.9	8	100
Propazine	C_9_H_16_ ClN_5_		10.62	230.1	104	35	100
Sebuthylazine	C_9_H_16_ClN_5_		10.85	230.12	174.05	16	135
Simazine	C_7_H_12_ClN_5_		7.87	202.1	104	20	120
			7.87	202.1	71.1	20	120
Terbutylazine	C_9_H_16_ClN_5_		10.85	230.1	96.1	40	70
			10.85	230.1	68	24	70

Standard references for LC–MS/MS analysis, including atrazine-desethyl, cyanazine, simazine, atrazine, propazine, sebuthylazine, deisopropylatrazine, terbuthylazine, 2,4 dichlorophenoxyacetic acid (2,4-D), diuron, and linuron (with a purity > 99%), were obtained from CPAchem Ltd. (Bogomilovo, Bulgaria). Additionally, a mixed solution of 16 EPA-PAHs was procured from AccuStandard (Connecticut, USA) in a dichloromethane solution at a concentration of 200 mg/L.

### Open burn test facility

Rice straw biomass was subjected to controlled burning following a study of Junpen et al.^4^ within an open burn test facility located in Sanpatong district, Chiang Mai, Thailand (18.6385° N, 98.8367° E). The burning test was performed following a study by Junpen et al.^4^ with modifications. The burning test area measured 30 × 30 m^2^. However, due to strict measures in Chiang Mai province to control burning by issuing bans, the experiment could not be conducted on a larger scale. These measures aimed to mitigate the environmental and health impacts of burning agricultural residues and forest fires. The experimental site's flat topography resulted in fluctuating wind flow rates between 8.0 and 11.3 km/h, leading to variable wind directions during the burning tests. The ambient temperature ranged from 27 to 32 °C.

### Burning experiment

**Figure 1:**
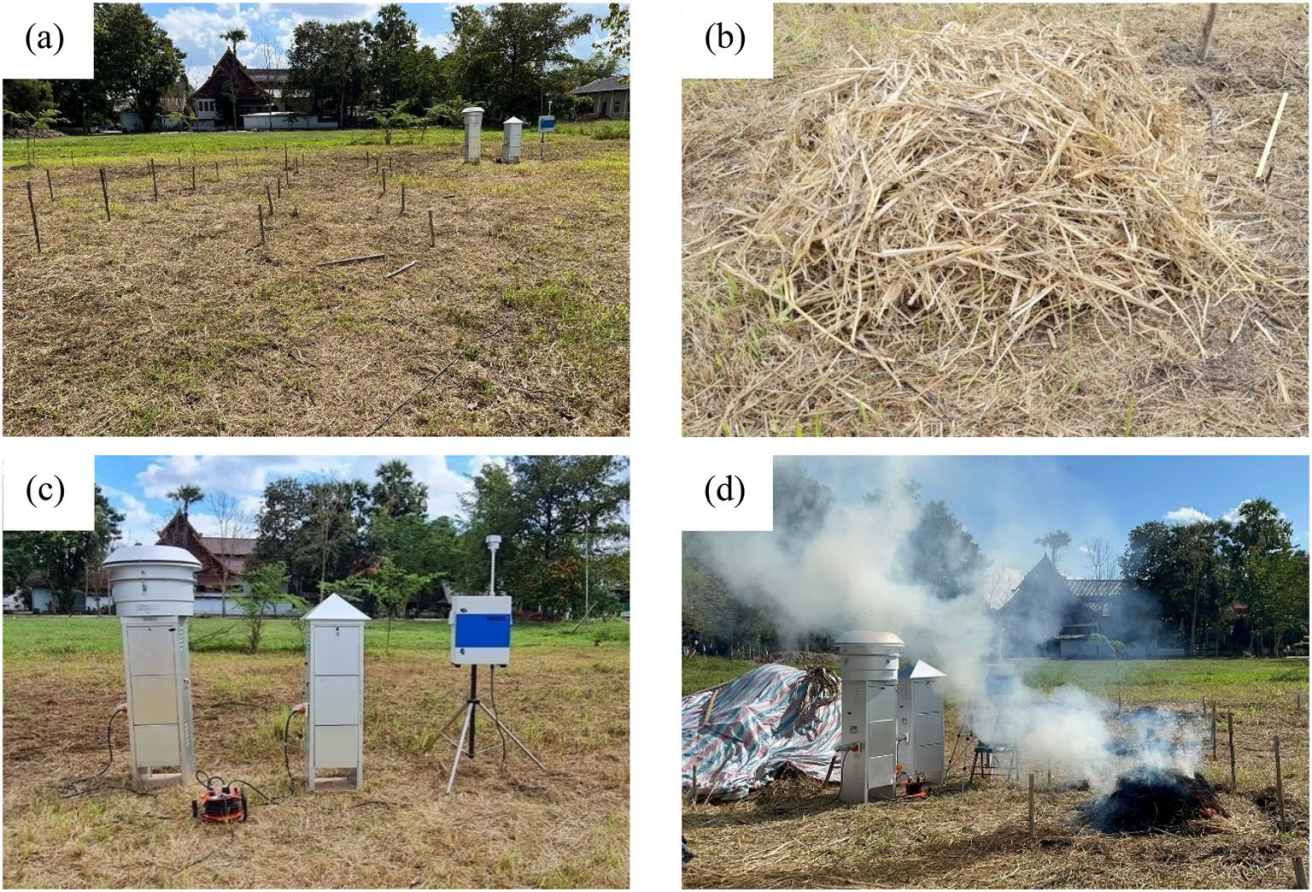
Open burning experiment: (**a**) area preparation; (**b**) preparation of rice straw biomass before burning; (**c**) preparation of air sampling instruments; and (**d**) air sampling during incomplete combustion.

This experiment was conducted in November 2022. An amount of approximately 20 kg of rice straw biomass was prepared, with dimensions measuring 1.0 × 1.0 × 0.5 m^3^ (width, length, and height). The biomass was evenly coated with a hand sprayer and allowed to air-dry outdoors overnight to ensure optimal conditions. To ensure consistency and minimize the effects of seasonal variations, the entire test program was limited to four days. The rice straw biomass was subjected to herbicide treatment, in accordance with the recommended concentrations by the manufacturer for paddy field applications. Specifically, ATZ was applied at a concentration of 540 mg/kg, and DIU was administered at 480 mg/kg. Each 500 ml of the respective herbicide was meticulously sprayed onto the 20 kg of biomass. The ignition process was initiated at a single point, strategically positioned at the center of the base. The ignition typically required approximately 5 s to facilitate the establishment of self-sustained combustion. 6 The entire burning process, as illustrated in Fig. 1, was replicated three times for each herbicide. Prior to ignition, soil, biomass, and air samples were collected to establish baseline environmental conditions, serving as vital references for subsequent analyses. Tests were conducted with a minimum 8 m separation between areas. Methodological precision was ensured with two random burning tests daily at 8 am and 1 pm. Precautionary measures, including consistent plastic sheeting, were in place to prevent unused straw biomass contamination.

### Sampling procedure

#### Sampling of soil and biomass residues

Collections of topsoil samples, originating from depths of 0–15 cm, were conducted both before and after the open burning test. These sampling procedures entailed gathering soil from three randomized positions within each designated area, resulting in composite samples weighing approximately 500 g each. In parallel, samples of rice straw, amounting to around 100 g, were obtained prior to combustion, alongside ash residues generated during the burning experiments. All collected samples, encompassing both soil and biomass residues, were meticulously placed within amber reclosable zip-lock bags. Each bag was meticulously labeled for precise identification, and the samples were then swiftly transported to the laboratory. Subsequently, these samples were stored at room temperature, approximately 20–24 °C, for a 7-day equilibration period before undergoing further extraction and analytical procedures.

#### Sampling of air

In this study, air samples were collected under two conditions: during biomass burning and in the background atmosphere without burning. Total suspended particulate (TSP) samples were precisely collected using 20.3 × 25.4 cm glass fiber filters (ADVANTEC, Tokyo, Japan), while PM_10_ was gathered using 20.3 × 25.4 cm quartz–fiber filters (ADVANTEC, Tokyo, Japan). Both samples were acquired using a high-volume sampler following the protocols specified in the US EPA Federal Reference method IO-2.1^36^. To simulate the inhalation route of Thai residents, the sampler was positioned at a height of approximately 1.6 m and located at a distance of around 1.5 m from the burning area. Sampling was performed over a 30-min duration at an average flow rate of 1.1 m^3^/min, which corresponded to total sampled air volume of 33.9 m^3^. Following the completion of the sampling process, the filters were carefully retrieved and shielded in aluminium foil for preservation. Samples were then sealed in amber zip-lock bags, transported cool to the laboratory and stored in a freezer at a temperature of − 20 °C until the commencement of the analysis.

Aerosol samples were meticulously acquired using low-volume samplers, following the established procedures outlined in the US EPA Federal Reference method TO-10A^37^. The samplers were positioned at a height of approximately 1.6 m from the ground. These samplers operated for an average of 30-min intervals, maintaining a flow rate of about 1.1 m^3^/min. To collect both particles and gases simultaneously, a 75 mm-long glass tube (ORBO™ 49P OSHA Versatile Sampler, Merck KGaA, Darmstadt, Germany) with a 13-mm outer diameter at the inlet end tapered to 8 mm at the outlet end was employed, followed by specially cleaned XAD®-2 material (Merck KGaA, Darmstadt, Germany) positioned within a tube.

To ensure the representativeness of air samples, meticulous planning and execution were employed. Despite challenges posed by changing airflow, wind speed, and direction in an open environment, a systematic sampling strategy was utilized. Additionally, calibration of all sampling equipment was conducted according to manufacturer guidelines, along with field validation tests to ensure equipment performance under various conditions. Quality control measures such as triplicate samples, field blanks, and equipment blanks were implemented to assess reproducibility and monitor for contamination. After sampling and before the extraction process, the filters and adsorbents were carefully placed in clean amber zip-lock bags and stored in a dark environment at − 20 °C. The efficacy of this cleaning procedure was confirmed through the use of blank samples.

### Extraction methods

#### Extraction of soil and biomass residues

All samples underwent extraction following the QuEChERS method, as described by Valverde et al.^38^ with some modifications. Approximately 10 g of soil or 1 g of biomass was accurately weighed and placed in a 50-mL centrifuge tube. Subsequently, 10 mL of water was introduced into the tube, and the mixture was allowed to stand for 30 min at ambient temperature. Following this, 10 mL of acetonitrile were added to the tube. The tube was sealed, vigorously shaken by hand for 1 min, and a buffer-salt mixture was introduced to facilitate phase separation and herbicide partitioning. This buffer-salt mixture consisted of 4 g of magnesium sulfate anhydrous (MgSO_4_), 1 g of sodium chloride and 0.5 g of disodium hydrogen citrate sesquihydrate. The tube was resealed, shaken vigorously by hand for an additional minute, and then centrifuged using Multi Centrifuge (VARISPIN 4, NOVAPRO Co., Ltd., Seoul, Republic of Korea) at 4000 rpm for 5 min. Following this, 1 mL of the acetonitrile phase was transferred into a separate centrifuge tube containing 150 mg of MgSO_4_ and 25 mg of primary-secondary amine (PSA) to remove polar organic acids, some sugars, and lipids. The tube was sealed, shaken vigorously by hand for 30 s, and then centrifuged at 4000 rpm for 5 min using a Microliter and Haematocrit Centrifuge (NF 480, NÜVE SANAYİ MALZEMELERİ İMALAT VE TİCARET A.Ş, Ankara, Turkey). The residue was reconstituted with 0.5 mL of acetonitrile. Each final extract was then filtered through a 0.2 µm membrane filter into a 2 mL amber glass vial. These vials were stored at − 20 °C until LC–MS/MS analysis.

#### Extraction of particulate and aerosol samples for herbicides determination

For TSP and PM_10_, the filters containing samples were first cut into small pieces and then accurately weighed, with each sample weighing approximately 5 g, before being placed in 50-mL centrifuge tubes. The subsequent extraction process was carried out using the QuEChERS method as previously described.

The extraction of aerosol samples from the combined XAD®-2 material and filter was performed using a Soxhlet extraction method, in accordance with the US EPA Federal Reference method TO-10A^37^, with a slight modification. The extraction process involved the use of 100 mL of ethyl acetate as the solvent and an extraction time of 8 h, which were pre-determined. After undergoing a specialized Soxhlet extraction (Lab Valley Co., Ltd., Bangkok, Thailand), the resulting solution was further adjusted to a final volume of 2 mL before being injected into 2 mL chromatography vials. These vials were stored at − 20 °C before injection into the chromatographic system.

#### Extraction of particulate samples for PAHs determination

The extraction of PAHs in TSP and PM10 was conducted according to the method outlined by Wiriya et al.^39^ with slight modifications. For each sample, three pieces of 20 mm filters from TSP and PM_10_ collections were placed into a 20 mL glass vial. An equal amount of 20 µL mixed internal standard of PAHs, acenaphthene D10, perylene D12 (Dr. Ehrenstorfer, LGC, Augsburg, Germany), and 10 mL dichloromethane were added. Ultrasonic Cleaner S 30H (ELMA—Hans Schmidbauer GmbH & Co, Singen, Germany) at 10 °C for 10 min. The extract was filtered by a PTFE syringe filter (13 mm, 0.2 μm) and subsequently concentrated by concentrated by Buchi Heating bath B-491/Buchi Rotavapor R-210 (BUCHI Labortechnik AG, Flawil, Switzerland) at 35 °C to reduce the sample volume to approximately 0.5 mL. The extracts were then adjusted in volume to 1 mL with ethyl acetate before GC–MS analysis.

### Chromatographic determination

#### LC–MS/MS analysis for herbicides

The sample analysis was conducted using a method previously described by Blanchoud et al.^40^ with some modifications. The LC–MS/MS system used in this analysis comprised a 1290 vialsampler (G7129B, Agilent, California, USA), a 1290 high speed pump (G7129A, Agilent, California, USA), and a 1290 MCT detector (G7166B, Agilent, California, USA). Separation was carried out on a Phenomenex Luna C18(2) column (150 mm × 2.00 mm ID, particle size 5 µm). The eluents consisted of two components: (A) water containing 0.1% formic acid and 5 mM ammonium acetate (CH_3_CO_2_NH_4_), and (B) methanol containing 0.1% formic acid and 5 mM CH_3_CO_2_NH_4_. The flow rate was set at 0.3 mL/min. The gradient conditions were as follows: 0–2.5 min, a linear transition from 10 to 40% B; 2.5–12 min, a linear transition from 40 to 95% B; 12–13 min, a linear transition from 90 to 10% B. The autosampler and column temperatures were maintained at 4 and 40 °C, respectively, with a 2 µL injection volume. Detection of all chemical substances was performed using Agilent Jet Stream electrospray ionization (ESI) in the positive mode. The optimized operating conditions were as follows: capillary voltage ranged from 3200 to 3800 V, nebulizer pressure at 45 psi, sheath gas temperature at 400 °C with a sheath gas flow of 12 L/min, and gas temperature at 300 °C with a gas flow of 3 L/min. Collision gas pressure and tube lens offset voltages were optimized for each herbicide using the automated optimization procedure in syringe infusion mode provided by the manufacturer. The mass spectrometry scanning method utilized dynamic Multiple Reaction Monitoring (MRM). The monitored transitions for each herbicide are detailed in Table 2.

To ensure the suitability of the optimized procedure for routine application, a validation process was conducted, covering selectivity, linearity, limit of detection (LOD), and limit of quantification (LOQ). Selectivity was assessed using standards of various herbicides on the LC system, comparing the results with an aqueous solution of the analytes near the LOQs. No significant interference was detected in the retention time of the compounds, indicating good selectivity. Linearity was established through a linear regression approach, analyzing duplicate injections of herbicide standards at varied concentrations. The method exhibited significant linear regression (p < 0.05) across the evaluated concentration ranges, supported by high determination coefficient values (R^2^ ≥ 0.998). LODs and LOQs were determined following IUPAC Harmonized Guidelines, with LODs ranging from 0.00 to 7.42 ng/mL and LOQs from 0.00 to 22.83 ng/mL.

Recovery studies were evaluated from spiked samples, with values ranging from 90 to 95%, suggesting approximately 5–10% herbicide losses during the analytical procedure. Extraction recoveries were calculated by comparing the response of each analyte in the matrix-matched calibration curve with the response detected in the spiked samples after extraction. The average recovery values for both spike levels were higher than 80% for all analytes included in the study. Intra and inter-day precision (repeatability/reproducibility) was calculated for each analyte from results obtained from the recovery study in terms of relative standard deviations (RSD). In both cases, RSDs were within an acceptable limit of 5%.

#### GC–MC analysis for PAHs

To analyze PAHs contamination in TSP and PM10 samples, the procedure followed was similar to that described by Wiriya et al.^39^ with some modifications. A gas chromatography-mass spectrometry (GC–MS) using an Agilent 7890A gas chromatograph (Agilent, California, USA) was carried out. A capillary column (30 m × 0.25 mm × 0.25 μm; Agilent, California, USA) was employed. Helium served as the carrier gas, flowing steadily at a rate of 1.0 mL/min. An injection volume of 1 μL was used in spitless mode, while the injector temperature was maintained at 275 °C. The temperature of the oven followed a specific program: it initiated at 70 °C for 0.5 min, then increased at a rate of 20 °C/min to 150 °C, further increasing at 10 °C/min to 285 °C, and ultimately reaching 310 °C, where it was held for 6 min. The analysis was completed in a total time of 27.75 min. GC–MS analysis was conducted using electron impact ionization at 70 eV, and the ion source operated at 250 °C. To confirm the identity of the compounds in the samples, the selected reaction monitoring (SRM) mode was employed. The concentrations of PAHs were determined by constructing a 7-point calibration curve with deuterated PAHs serving as surrogate internal standards. The concentration of each compound was expressed in ng/m^3^.

LODs and LOQs for each analyte were determined following established methods with some modifications^39,41,42^. The LODs for the PAHs ranged from 0.29 ng/mL for anthracene to 1.42 ng/mL for chrysene, while the LOQs ranged from 0.97 ng/mL for anthracene to 4.74 ng/mL for chrysene. To determine the recoveries of individual PAHs, filter samples were spiked with PAHs (N = 7, 2.00 ng/mL each individual PAH). The mean recoveries ranged from 0.92 ± 0.20 ng/mL (46% recovery) for indeno[1,2,3-cd]pyrene to 3.20 ± 0.21 ng/mL (160% recovery) for acenaphthylene. The validation results affirm the presence of a significant linear regression (p < 0.05) within the examined concentration ranges, with strong determination coefficient values (R^2^ ≥ 0.998). These results underscore the precision and dependability of the extraction and analytical methodology in quantifying PAHs concentration in the air samples.

## Results

### Herbicide contamination in soil pre- and post-burning

Table 3 details the identified herbicides and their average concentrations in soils before and after burning herbicide-contaminated biomass. Initially, raw soil contained 0.10 mg/kg (79.2%) of 2,4-D and 0.03 mg/kg (20.8%) cyanazine. Following the burning of biomass contaminated with ATZ, five out of the eleven studied herbicides were detected. While 2,4-D remained the predominant compound, constituting 50.2% of the soil samples, its concentration experienced a significant 3.3 times decrease compared to raw soil. In addition, there was also a decrease in cyanazine concentrations. Remarkably, some chemical compounds, including atrazine-desethyl, sebuthylazine, and terbuthylazine, which were not present in the raw soil, were identified after the burning process. Conversely, burning biomass contaminated with DIU resulted in 2,4-D and cyanazine representing approximately 99.2% and 0.8%, respectively, of the detected herbicides in soils. Compared to raw soil, the concentration of 2,4-D in DIU-contaminated soils decreased by 3.4 times after burning, while cyanazine experienced a substantial 113.6 times decrease.

**Table 3:** Comparing herbicide concentrations in soil before and after biomass burning with atrazine and diuron contamination.

Identified herbicides	Concentration (mg/kg)		
	Raw soil before addition of ATZ or DIU (N = 3)	Atrazine added (N = 3)	Diuron added (N = 3)
Atrazine-desethyl	ND^1^	0.011 (0.004)	ND
Cyanazine	0.027 (0.008)	0.012 (0.001)	0.000 (0.000)
Simazine	ND	ND	ND
Atrazine	ND	ND	ND
Propazine	ND	ND	ND
Sebuthylazine	ND	0.003 (0.000)	ND
Deisopropylatrazine	ND	ND	ND
Terbuthylazine	ND	0.004 (0.006)	ND
2,4-D	0.102 (0.001)	0.031 (0.007)	0.030 (0.009)
Diuron	ND	ND	ND
Linuron	ND	ND	ND

All values show the mean (standard deviation).

^1^*ND* Not detected.

### Herbicide contamination in rice straw biomass pre- and post-burning

Table 4 illustrates the presence and concentrations of herbicides in raw and burned rice straw biomass contaminated with herbicides. In the untreated straw biomass, terbuthylazine dominated, comprising 80.0% of the total concentration, followed by 2,4-D at 18.4%, and cyanazine at 1.6%. After treating the straw biomass with ATZ before burning, ATZ was the main detected compound at 96.0%, followed by propazine at 2.3%, and terbuthylazine at 1.1%. Following the burning of ATZ-contaminated biomass, terbuthylazine, atrazine-desethyl, and 2,4-D, which were previously present in treated biomass with higher concentrations, decreased by 2.1, 1.7, and 2.4 times, respectively. Sebuthylazine exhibited a notable increase of 21.8 times compared to the treated biomass before burning. Simazine, ATZ, propazine, and deisopropylazine, which were found at high concentrations in straw biomass treated with ATZ, disappeared after burning.

**Table 4:** Comparing herbicide concentrations in straw biomass before and after burning with atrazine and diuron contamination.

Identified herbicides	Concentration (mg/kg)				
	Before burning			After burning	
	Raw biomass before addition of ATZ or DIU (N = 3)	Atrazine added (N = 3)	Diuron added (N = 3)	Atrazine added (N = 3)	Diuron added (N = 3)
Atrazine-desethyl	ND^1^	0.248 (0.006)	ND	0.148 (0.020)	ND
Cyanazine	0.083 (0.007)	0.083 (0.005)	0.091 (0.005)	0.087 (0.005)	0.079 (0.011)
Simazine	ND	5.282 (0.120)	ND	ND	ND
Atrazine	ND	1155.77 (18.35)	0.915 (0.003)	ND	ND
Propazine	ND	27.285 (1.023)	ND	ND	ND
Sebuthylazine	0.003 (0.004)	0.004 (0.002)	0.004 (0.002)	0.087 (0.016)	ND
Deisopropylatrazine	ND	0.826 (0.010)	ND	ND	ND
Terbuthylazine	4.114 (0.667)	12.657 (0.828)	4.262 (0.014)	6.119 (0.040)	ND
2,4-D	0.946 (0.050)	1.254 (0.025)	1.108 (0.005)	0.512 (0.204)	0.666 (0.050)
Diuron	ND	ND	1090.93 (10.26)	ND	ND
Linuron	ND	ND	ND	ND	ND

All values show the mean (standard deviation).

^1^*ND* Not detected.

After the application of DIU, straw biomass before burning contained 99.4% DIU, followed by 0.4% terbuthylazine, and 0.1% 2,4-D. Interestingly, in burned biomass contaminated with DIU, 2,4-D dominated, accounting for 89.4% of the total concentration, followed by cyanazine at 10.6%. The concentrations of 2,4-D and cyanazine decreased by 1.7 and 1.2 times, respectively, after the burning of DIU-contaminated biomass. ATZ, DIU, sebuthylazine, and terbuthylazine, present in raw samples after DIU application, were no longer detectable following burning with DIU contamination.

### Herbicide contamination in air pre- and post-burning

The identified herbicides and their average concentrations in TSP are outlined in Table 5. Before the burning process, six out of eleven herbicides were detected in ambient TSP, with linuron exhibiting the highest concentration (72.2%), followed by ATZ (14.7%), and DIU (11.8%). After burning ATZ-contaminated biomass, nine herbicides were observed. Although linuron remained the dominant compound (43.9%), its concentration decreased by 1.6 times compared to the background sample. Atrazine levels were significantly elevated, increasing by 2.9 times from the background. While cyanazine and atrazine-desethyl exhibited slight increases after burning, the concentrations rose by 33.4 and 4.1 times, respectively. In the case of DIU application in burned biomass, the concentration of linuron notably decreased by 4.6 times, while diuron increased by 7.1 times compared to the background. Atrazine-desethyl, cyanazine, and deisopropylatrazine were no longer detected in the TSP following DIU application.

**Table 5:** Comparing herbicide concentrations in total suspended particulate (TSP) before and after burning biomass with atrazine and diuron contamination.

Identified herbicides	Concentration (mg/kg)		
	TSP before burning (N = 3)	Atrazine added (N = 3)	Diuron added (N = 3)
Atrazine-desethyl	0.266 (0.0134)	1.129 (0.371)	0.142 (0.062)
Cyanazine	0.036 (0.004)	1.240 (0.269)	0.030 (0.002)
Simazine	ND^1^	0.025 (0.036)	ND
Atrazine	6.225 (0.064)	18.501 (0.574)	3.459 (0.072)
Propazine	ND	ND	ND
Sebuthylazine	ND	0.144 (0.015)	ND
Deisopropylatrazine	0.256 (0.019)	0.118 (0.005)	0.013 (0.019)
Terbuthylazine	ND	ND	ND
2,4-D	ND	0.033 (0.046)	0.347 (0.014)
Diuron	4.999 (1.045)	3.580 (0.559)	275.903 (74.128)
Linuron	30.643 (3.379)	19.349 (0.980)	51.496 (6.091)

All values show the mean (standard deviation).

^1^*ND* Not detected.

The herbicide profile in PM_10_, both before and after biomass burning with herbicide contamination, is outlined in Table 6. In ambient PM_10_, linuron and atrazine were predominant, accounting for 50.2% and 48.3%, respectively. Following the burning of ATZ-contaminated biomass, ATZ levels increased approximately 1.6 times, while linuron decreased by 2.6 times compared to ambient PM_10_ (background). Deisopropylatrazine was not detected in PM_10_ after ATZ application, but cyanazine and atrazine-desethyl emerged. In the case of DIU application, linuron, though detected at the highest concentration, increased only 1.3 times from the background, while DIU levels surged by 15.7 times. ATZ, present in the background, decreased after the burning process.

**Table 6:** Comparing herbicide concentrations in particulate matter (PM_10_) before and after burning biomass with atrazine and diuron contamination.

Identified herbicides	Concentration (mg/kg)		
	PM_10_ before burning (N = 3)	Atrazine added (N = 3)	Diuron added (N = 3)
Atrazine-desethyl	0.134 (0.033)	0.685 (0.004)	0.047 (0.014)
Cyanazine	0.038 (0.001)	3.313 (0.003)	0.016 (0.023)
Simazine	ND^1^	ND	ND
Atrazine	161.293 (7.620)	424.687 (65.647)	43.249 (4.980)
Propazine	ND	ND	ND
Sebuthylazine	ND	0.052 (0.001)	0.005 (0.000)
Deisopropylatrazine	0.376 (0.064)	ND	ND
Terbuthylazine	ND	1.173 (0.371)	0.004 (0.001)
2,4-D	ND	ND	0.228 (0.096)
Diuron	4.306 (0.375)	3.334 (0.863)	60.589 (14.428)
Linuron	167.450 (3.560)	106.013 (0.297)	195.249 (3.790)

All values show the mean (standard deviation).

^1^*ND* Not detected.

Table 7 presents the influence of the burning process on the type and concentration of herbicides detected in aerosols. Atrazine was the sole herbicide identified in ambient aerosol before biomass burning. Following the burning of ATZ-contaminated biomass, the concentration of atrazine increased by approximately 3.3 times, accompanied by the appearance of sebuthylazine and terbuthylazine, constituting around 3.1% and 2.4% of all detected herbicides. In the case of aerosol with DIU application in burned biomass, atrazine was the only herbicide detected, albeit at a lower concentration, decreased by 3.7 times from the background sample. Notably, none of the phenylurea herbicides, DIU, and linuron were detected in any aerosol samples.

**Table 7:** Comparing herbicide concentrations in aerosol before and after burning biomass with atrazine and diuron contamination.

Identified herbicides	Concentration (mg/kg)		
	Aerosol before burning (N = 3)	Atrazine added (N = 3)	Diuron added (N = 3)
Atrazine-desethyl	ND^1^	ND	ND
Cyanazine	ND	ND	ND
Simazine	ND	ND	ND
Atrazine	0.020 (0.003)	0.066 (0.006)	0.005 (0.008)
Propazine	ND	ND	ND
Sebuthylazine	ND	0.002 (0.003)	ND
Deisopropylatrazine	ND	ND	ND
Terbuthylazine	ND	0.002 (0.002)	ND
2,4-D	ND	ND	ND
Diuron	ND	ND	ND
Linuron	ND	ND	ND

All values show the mean (standard deviation).

^1^*ND* Not detected.

Table 8 displays the presence and concentrations of PAHs in TSP before and after the combustion of biomass contaminated with herbicides. Prior to burning, ambient TSP (background) already contained eight out of the eleven types of PAHs, with four of them—BaA, BbF, Ant, and DahA—identified as mutagenic or carcinogenic compounds. Post-burning of herbicide-contaminated biomass, all studied PAHs were detected, and their concentrations exhibited an increase. Specifically, BaA, BaP, and BbF emerged as the three predominant carcinogenic PAHs after burning biomass contaminated with both ATZ and DIU. Following the burning of ATZ-contaminated biomass, BaA, BaP, and BbF concentrations rose approximately 9.9, 13.9, and 9.2 times, respectively, compared to ambient TSP. Similarly, in the case of DIU-contaminated biomass burning, concentrations increased by 4.7 times for BaA, 10.0 times for BaP, and 6.6 times for BbF. Additionally, carcinogenic PAHs such as Chr and IcdP, absent in ambient TSP, were detected after burning biomass contaminated with both ATZ and DIU.

**Table 8:** Polycyclic aromatic hydrocarbons (PAHs) concentration in total suspended particulate (TSP) samples with herbicide-enhanced burned biomass.

Identified PAHs	Concentration (ng/m^3^)		
	TSP before burning (N = 3)	Atrazine added (N = 3)	Diuron added (N = 3)
Acenaphthylene (Acy)	1.022 (0.486)	1.572 (0.350)	1.342 (0.012)
Acenaphthene (Ace)	2.131 (1.032)	2.791 (0.265)	2.932 (0.056)
Fluorine (Flu)	0.250 (0.123)	0.414 (0.187)	0.304 (0.028)
Anthracene (Ant)	0.772 (0.371)	1.172 (0.234)	1.182 (0.324)
Benzo[a]anthracene (BaA)*	0.571 (0.282)	5.652 (6.953)	2.701 (3.357)
Chrysene (Chr)*	ND^1^	4.105 (8.231)	1.710 (4.414)
Benzo[b]fluoranthene (BbF)*	0.333 (0.160)	3.051 (3.092)	2.182 (2.862)
Benzo[a]pyrene (BaP)*	0.314 (0.145)	4.320 (5.252)	3.094 (4.312)
Indeno[1,2,3-cd]pyrene (IcdP)*	ND	0.982 (1.151)	0.165 (0.773)
Dibenz[a,h]anthracene (DahA)*	1.002 (0.472)	2.051 (1.052)	1.363 (0.120)
Benzo[g,h,i]perylene (BghiP)	ND	0.774 (0.960)	0.052 (0.461)

*Carcinogenic PAHs.

All values show the mean (standard deviation).

^1^*ND* Not detected.

Table 9 presents the concentrations of the investigated PAHs in PM_10_ samples before and after the burning of biomass contaminated with herbicides. Notably, the variety of PAHs found in PM_10_ was greater than that in TSP. In ambient PM_10_ (background), eight out of thirteen types of PAHs were detected, with four of them identified as carcinogenic compounds. Following the burning of ATZ-contaminated biomass, the concentrations of BaA, BaP, and Chr emerged as the top three dominant PAHs in PM_10_. Intriguingly, the concentrations of BaA and BaP increased by approximately 11.4 and 19.0 times, respectively, compared to the background sample. While non-carcinogenic PAHs Ace, Acy, and Ant were the main PAHs identified in PM_10_ after burning DIU-contaminated biomass, a noticeable decrease in carcinogenic PAHs BaP and BbF was observed compared to the background sample. It is important to highlight that Fiu, Chr, BkF, IcdP, and BghiP, undetected in both the background and PM_10_ after burning DIU-contaminated biomass, were found in PM_10_ after burning ATZ-contaminated biomass, with three of them identified as carcinogenic PAHs.

**Table 9:** Polycyclic aromatic hydrocarbons (PAHs) concentration in particulate matter (PM_10_) samples with herbicide-enhanced burned biomass.

Identified PAHs	Concentration (ng/m^3^)		
	PM_10_ before burning (N = 3)	Atrazine added (N = 3)	Diuron added (N = 3)
Acenaphthylene (Acy)	1.062 (0.502)	1.361 (0.052)	1.742 (0.721)
Acenaphthene (Ace)	2.364 (1.262)	3.022 (0.314)	2.770 (0.032)
Fluorine (Flu)	0.240 (0.123)	0.730 (0.792)	0.292 (0.044)
Anthracene (Ant)	0.772 (0.361)	1.414 (0.524)	1.132 (0.192)
Fluoranthene (Fiu)	ND^1^	0.752 (2.464)	ND
Benzo[a]anthracene (BaA)*	0.553 (0.243)	6.282 (8.582)	0.783 (0.090)
Chrysene (Chr)*	ND	5.384 (10.572)	ND
Benzo[b]fluoranthene (BbF)*	0.343 (0.172)	3.645 (4.113)	0.791 (0.311)
Benzo[k]fluoranthene (BkF)*	ND	0.281 (3.290)	ND
Benzo[a]pyrene (BaP)*	0.311 (0.141)	5.880 (6.790)	0.904 (0.502)
Indeno[1,2,3-cd]pyrene (IcdP)*	ND	0.912 (1.582)	ND
Dibenz[a,h]anthracene (DahA)*	1.002 (0.470)	1.503 (0.211)	1.292 (0.002)
Benzo[g,h,i]perylene (BghiP)	ND	0.432 (0.851)	ND

*Carcinogenic PAHs.

All values show the mean (standard deviation).

^1^*ND* Not detected.

## Discussion

### Residual herbicides in soil and ash samples following burning

In this study, 2,4-D emerges as the predominant compound in both raw soil and biomass samples due to its widespread use as an herbicide in agriculture. Despite its relatively short aerobic soil half-life of approximately 6.2 days^43^, the persistence of 2,4-D bound residues is evident, linked to soil organic matter and its diverse sorption sites, as discussed by Boivin et al.^44^. In plants, 2,4-D salt formulations are mainly absorbed through roots, while esters are readily absorbed through foliage. Efficient translocation of foliar-applied 2,4-D occurs through the phloem, traveling from photosynthesizing leaves to growth sites where it accumulates. Root-mediated upward translocation mainly takes place in the xylem's transpiration stream^45^. These mechanisms contribute to the observed high accumulation of 2,4-D in raw biomass samples before the application of ATZ or DIU.

After the combustion of biomass contaminated with ATZ and DIU, a considerable reduction in the concentration of 2,4-D in both soil and biomass samples implies its degradation during the burning process. Muhammad et al.^46^ and Lawal et al.^47^ underscore the significant impact of temperature on 2,4-D breakdown, with higher temperatures (> 40 °C) accelerating degradation rates. Like other herbicides, the fate of 2,4-D residues in soil and biomass may undergo various processes, including runoff, adsorption, chemical and microbial degradation, photodecomposition, and leaching^48–50^. Meftault et al.^50^ emphasized that the groundwater ubiquity score (GUS), leachability index (LIX), and hysteresis index (HI) values calculated for all urban soils indicate that 2,4-D is highly mobile and more likely to leach from surface soil to groundwater. This movement could potentially impact drinking water quality, non-target organisms, and food safety. Additionally, 2,4-D from impermeable surfaces could serve as a direct source of surface and groundwater contamination. Therefore, caution should be exercised when applying 2,4-D, especially on impervious surfaces, as higher concentrations may pose a risk to the health of ecologically significant biota^48^.

The presence of atrazine-desethyl, sebuthylazine, and terbuthylazine in soils after the combustion of biomass contaminated with ATZ may be attributed to their initial presence in the pesticide mixture applied to the biomass or their degradation pathway^51^. The disappearance of main compounds such as ATZ or DIU in treated straw biomass, or the observed fluctuations in other specific substances in samples after burning, suggest complex chemical transformations and decomposition processes during burning. This indicates that these herbicides undergo significant changes in their chemical structure when exposed to high temperatures, leading to the formation of new compounds or the breakdown of existing ones.

The degradation pathways of ATZ and DIU due to thermal degradation remain incompletely understood. Only a study of Książczak et al.^52^ provided insights into the thermal decomposition pathway of ATZ, employing thermogravimetric analysis (TG). Their study revealed a three-stage degradation process for ATZ: alkyl group removal, ethyl group elimination to form ethylene, and chlorine removal. Similar steps were observed in the degradation of ATZ metabolites, leading to the formation of four decomposition products: de-ethyl-deisopropyl-atrazine (DEIA), desethyl-atrazine (DEA), desisopropyl-atrazine (DIA), and hydroxyatrazine. Amino groups within the triazine ring corresponded to a higher amount of non-volatile thermal degradation products. Chlorine substituents facilitated the formation of products with low volatility, and hydroxyatrazine underwent further conversion to N-isopropylammelide or N-ethylammelide intermediates through hydrolytic deamination reactions catalyzed by amidohydrolases. Lu et al.^53^ explored the impact of heat/peroxymonosulfate (PMS) on ATZ degradation, proposing potential degradation products. For DIU, Gomez et al.^54^ conducted a comprehensive study on DIU thermal decomposition by pyrolysis at temperatures ranging from 400 to 1000 °C in a helium atmosphere. They found that dimethylamine was the sole amine detected during DIU pyrolysis. Additionally, the pyrolysis process of DIU resulted in the emission of CO, hydrogen cyanide (HCN), and hydrogen chloride (HCl). The initial breakdown involved the cleavage of N-CO bonds, producing isocyanate and amine, which further degraded into substituted ureas and various gaseous products. Other two primary degradation pathways have been also proposed: photo-transformation facilitated by sunlight and biodegradation by soil microorganisms, with the predominant compound resulting from DIU transformation being 3,4-dichloroaniline (3,4-DCA).

Biomass combustion could generate a diverse array of chemical compounds, subject to oxidation or direct emission into the atmosphere^55^. Bush et al.^56^ suggested that temperatures above 500 °C facilitate complete decomposition of most herbicides and insecticides, while smoldering temperatures below 500 °C may only partially decompose some herbicides, volatilizing significant proportions. Temperature alone is not the sole factor influencing herbicide decomposition, as evidenced by the presence of certain herbicides like carbamate in char resulting from wood pellet combustion^55^. It is important to note that the burning rate is a critical factor in thermal degradation, regardless of whether the atmosphere is inert or oxidizing. This observation not only sheds light on the decomposition mechanisms but also emphasizes the potential for undecomposed residues from ATZ or DIU products to adhere to soil or biomass samples. This highlights the need for careful consideration of burning conditions and their effects on the fate of herbicide residues during biomass burning processes. Previous studies^57,58^ have indicated that the residues of these herbicides have the potential to migrate and contaminate nearby areas, potentially harming surface and groundwater quality.

### Residual herbicides in air samples following burning

Biomass burning is well-known for releasing substantial amounts of PM, black carbon (BC), and toxic gases like carbon monoxide (CO), carbon dioxide (CO_2_), methane (CH_4_), nitrous oxide (N_2_O), volatile organic compounds (VOCs), semi-volatile organic compounds (SVOCs), and chlorofluorocarbons (CFCs) into the atmosphere, as extensively documented in previous studies^4,59^. Despite this, prior research has largely overlooked the potential contamination of herbicides in these particulate emissions during open burning. This study reveals the significant presence of various herbicides in TSP, PM_10_, and aerosols following the burning of straw biomass. Furthermore, noticeable changes in herbicide concentrations were observed in TSP, PM_10_, and aerosols when biomass was burned with applications of ATZ and DIU. These fluctuations can be attributed to the complex interplay of thermal decomposition, transformation, or reduced volatility during the combustion process.

A comparison with other studies reveals both commonalities and unique compounds in the degradation products of ATZ and DIU in air samples. The diverse range of transformation and decomposition products observed may stem from the absence of prior research on the influence of open burning or combustion processes on ATZ and DIU herbicides in the atmosphere. This variability is likely impacted by the elevated temperature and abundant oxygen conditions inherent in the open burning process. Notably, this study included additional herbicides like propazine, terbuthylazine, and simazine in ATZ, and ATZ in DIU, adding complexity to the composition and influencing the formation and degradation processes of specific compounds found in TSP, PM_10_, and aerosol. This aligns with Chen et al.^60^, who highlighted that toxic product formation depends on fire conditions and material response, emphasizing the potential for incomplete combustion to generate more toxic species due to the fragmentation and rearrangement of herbicide structures involving sulfur, nitrogen, phosphorus, and chlorine.

The observed results highlight that primary herbicides and their transformed products exhibit a stronger tendency to adhere to PM, particularly TSP and PM_10_, as opposed to the broader aerosol category. This affinity is attributed to the solid particles in TSP and PM_10_, providing additional surfaces for herbicide adsorption. While aerosols encompass particles of varied sizes, the unique characteristics of TSP and PM_10_ enhance their suitability for herbicide absorption^61,62^. In addition, the presence of ATZ in aerosol samples beyond PM_10_ and TSP, following herbicide-contaminated biomass burning, suggests distinctive affinities or behaviors of ATZ leading to its selective presence in the aerosol phase. This occurrence is likely influenced by factors like the compound's physical and chemical properties, volatility, and specific burning conditions, emphasizing the unique attributes of ATZ under these conditions and its preferential release into the aerosol phase.

Residual herbicides from biomass burning can contaminate the air extensively, potentially causing widespread environmental and health issues. ATZ and DIU have been linked to specific impacts on the development, reproduction, and behavior of various aquatic organisms^63^. This environmental presence poses a significant risk to aquatic microorganisms and fauna. Moreover, airborne exposure to these herbicides has been associated with acute and chronic health effects in individuals of all ages^64^. The toxic nature of airborne pesticides, coupled with the prolonged exposure individuals may experience, whether voluntarily or involuntarily, has been linked not only to an elevated incidence of cancer^65^ but also to potential disruptions of the endocrine and immune systems^66^. Despite the incomplete understanding of the precise mechanisms underlying these health impacts, compelling evidence indicates that herbicides can disrupt enzymatic function and signaling mechanisms at the cellular level^67^. Additionally, DNA-based toxicity research suggests that airborne herbicides may alter gene expression^68^, potentially leading to inherited epigenetic changes. These chemicals have been also linked to risks for the nervous system^16^. Given the potential environmental and health risks associated with residual herbicides from biomass burning, it is crucial to implement effective management strategies.

In the context of PAHs, the incomplete combustion of crop residues is a well-documented source of PAH emissions^69,70^. PAHs, recognized as harmful persistent organic pollutants, pose risks to the environment^71^, animals^72^, and public health^73^. This study reveals a significant increase in carcinogenic PAHs (BaA, BaP, BbF) in both TSP and PM_10_ samples after burning herbicide-contaminated biomass. The presence of ATZ and DIU in biomass could potentially alter the composition of PAHs through complex chemical interactions during burning. Previous research by Zhang et al.^74^ suggested that biomass with high volatile contents can generate abundant phenyl radicals during burning, leading to significant emissions of PM-bound PAHs. This finding may be particularly relevant for biomass containing volatile pesticides, indicating a potential mechanism for PAH release during combustion. Additionally, McGrath et al.^75^ demonstrated that low and medium-molecular-weight PAHs are predominantly emitted during biomass burning at temperatures equal to or higher than 400 °C, while high-molecular-weight PAHs are more likely to form at temperatures ≥ 500 °C. Moreover, the emission of PAHs tends to increase within a specific temperature range, with higher temperatures favoring the synthesis of PAHs from fragments produced by biomass pyrolysis during burning^74^. Regarding the role of oxygen supply, De Gennaro et al.^76^ emphasized its significance in influencing PAH emissions during biomass burning, showing that biomass burning in a fireplace led to higher PAH emissions compared to burning in a woodstove. While certain PAHs may not inherently possess carcinogenic properties, it is crucial to acknowledge that upon release into the atmosphere, gas-phase PAHs can undergo transformations, potentially forming more potent carcinogenic and mutagenic forms, such as nitro-PAHs^77^. Diverse interactions between PAHs and PM are suggested by their different behaviors in TSP and PM_10_, likely influenced by factors such as particle size, molecular weight, and composition^78^. Rice straw smoke PM, as noted by Woodrow et al.^79^, contains phenols and mutagens, presenting health risks comparable to those of cigarette smoke. The release of unknown quantities of toxic substances during unpredictable field and forest fires highlights the need for caution, particularly during atmospheric inversions, to restrict or prohibit open burning practices for the protection of public health.

## Conclusions

This study delved into the impact of burning herbicide-contaminated biomass on various environmental matrices, revealing substantial changes in herbicide distribution and concentrations. Notably, ATZ had a more pronounced negative impact than DIU. The combustion of ATZ-contaminated biomass produced diverse decomposition/transformation products (e.g., atrazine-desethyl, sebuthylazine, terbuthylazine) affecting both soil and biomass. In contrast, DIU primarily altered herbicide concentrations in burned residues. The investigation extended to atmospheric components, unveiling herbicide concentration shifts in TSP, PM_10_, and aerosol samples. ATZ demonstrated a notable tendency for aerosolization during biomass burning, raising concerns about potential health and environmental effects. Identifying carcinogenic PAHs in both TSP and PM_10_ after burning biomass contaminated with ATZ and DIU emphasized the complex interplay between herbicide contamination and hazardous pollutant generation. These findings underscore the need for thorough assessments and effective mitigation strategies in agricultural practices, emphasizing a comprehensive consideration of enduring health and environmental dynamics associated with ATZ, DIU, and their post-burning degradation/transformation products. Exploring holistic strategies is crucial for ensuring sustainable and environmentally responsible agricultural practices in the future.

## Data Availability

All data generated or analysed during this study are included in this published article.
